# Retinoic Acid Signalling Is Activated in the Postischemic Heart and May Influence Remodelling

**DOI:** 10.1371/journal.pone.0044740

**Published:** 2012-09-28

**Authors:** Dusan Bilbija, Fred Haugen, Julia Sagave, Anton Baysa, Nasser Bastani, Finn Olav Levy, Allan Sirsjö, Rune Blomhoff, Guro Valen

**Affiliations:** 1 Department of Physiology, University of Oslo, Oslo, Norway; 2 Department of Nutrition, Institute of Basic Medical Sciences, University of Oslo, Oslo, Norway; 3 Department of Pharmacology, Institute of Clinical Medicine, University of Oslo, Oslo, Norway; 4 Center for Heart Failure Research, University of Oslo, Oslo, Norway; 5 Division of Biomedicine, Department of Clinical Medicine, Örebro University, Örebro, Sweden; University of Cincinnati, United States of America

## Abstract

**Background:**

All-trans retinoic acid (atRA), an active derivative of vitamin A, regulates cell differentiation, proliferation and cardiac morphogenesis via transcriptional activation of retinoic acid receptors (RARs) acting on retinoic acid response elements (RARE).We hypothesized that the retinoic acid (RA) signalling pathway is activated in myocardial ischemia and postischemic remodelling.

**Methods and Findings:**

Myocardial infarction was induced through ligating the left coronary artery in mice. *In vivo* cardiac activation of the RARs was measured by imaging RARE-luciferase reporter mice, and analysing expression of RAR target genes and proteins by real time RT-PCR and western blot. Endogenous retinoids in postinfarcted hearts were analysed by triple-stage liquid chromatography/tandem mass spectrometry. Cardiomyocytes (CM) and cardiofibroblasts (CF) were isolated from infarcted and sham operated RARE luciferase reporter hearts and monitored for RAR activity and expression of target genes. The effect of atRA on CF proliferation was evaluated by EdU incorporation. Myocardial infarction increased thoracic RAR activity *in vivo* (p<0.001), which was ascribed to the heart through *ex vivo* imaging (p = 0.002) with the largest signal 1 week postinfarct. This was accompanied by increased cardiac gene and protein expression of the RAR target genes retinol binding protein 1 (p = 0.01 for RNA, p = 0,006 for protein) and aldehyde dehydrogenase 1A2 (p = 0.04 for RNA, p = 0,014 for protein), while gene expression of cytochrome P450 26B1 was downregulated (p = 0.007). Concomitantly, retinol accumulated in the infarcted zone (p = 0.02). CM and CF isolated from infarcted hearts had higher luminescence than those from sham operated hearts (p = 0.02 and p = 0.008). AtRA inhibited CF proliferation *in vitro* (p = 0.02).

**Conclusion:**

The RA signalling pathway is activated in postischemic hearts and may play a role in regulation of damage and repair during remodelling.

## Introduction

Retinoic acid metabolites, the active derivatives of vitamin A, are involved in tissue homeostasis in health and disease [1]. Retinoic acid (RA) orchestrates signal transduction pathways regulating embryonic development and cellular differentiation and proliferation [2]. Vitamin A is obtained from the diet as retinyl esters, or from provitamin A carotenoids as β-carotene, which are converted to retinol. Delivery of retinol to cells and its transformation into active retinoic acid metabolites is complex. It requires delivery of retinol by retinol binding proteins (RBPs), transport across the cell membrane by membrane receptor for plasma RBP (STRA6), synthesis of biologically active forms by metabolic enzymes (i.e. oxidation enzymes of the ALDH family) and delivery of metabolites to nuclear retinoic acid receptors by cellular binding proteins (CRBPs and CRABPs). Endogenous levels of RA are self-regulated by cytochrome P450 superfamily of enzymes (CYP26A1, CYP26B1 and CYP26C1), which convert all-trans RA (atRA) to hydroxylated inactive forms [3].

Heart failure is an increasing clinical challenge due to improved treatment of myocardial infarction and a steadily aging population. The process of remodelling may be initiated by myocardial injury such as infarction or pressure- or volume overload [4]. It is at first an adaptive response to maintain normal function, but when detrimental stimuli overpower adaptive capacity progressive decompensation follows. Remodelling is often associated with activation of fetal gene programs [4]. Our current understanding of the processes of remodelling and heart failure development is incomplete, and treatment regimens remain to be improved. Vitamine A may play a role in remodelling of the heart. During early embryogenesis RA orchestrates organogenesis and formation of the heart across various species [5]. Dietary intake of alpha- and beta-carotene reduced the risk of acute myocardial infarction in a case-control study of coronary artery disease patients [6]. In adult rats, vitamin A deficiency causes left ventricular dilatation resulting in a decline of cardiac function [7]. Evidence suggests that supplementation with atRA may prevent left ventricular dilatation and preserve ventricular function in rats with induced infarction [8]. RA may oppose various hypertrophic stimuli *in vitro* and preserve a normal phenotype of cardiomyocytes [9]. Thus, atRA may be a therapeutic candidate for the prevention and therapy of cardiac hypertrophy and remodelling in postnatal life. However, the endogenous expression pattern of RA target genes in the acute phase of infarction and in long term remodelling is not well characterized.

RA exists as the derivatives atRA, 13-cis RA or 9-cis RA. AtRA exerts its actions mainly through binding to the nuclear receptor RAR (α, β, or γ), while its enantiomer 9-cis RA binds to RAR or RXR (α, β, or γ), [10]. The receptors act as ligand-dependent transcription factors, and form heterodimers binding to promoter RAR elements (RARE) [11]. The heterodimers have two functions; modulate the frequency of transcription of target genes after binding to RARE, and cross-talk with other signalling pathways. RXR is a polyvalent cooperator for various nuclear receptors such as thyroid hormone receptors, vitamin D receptors, peroxisomal proliferator-activated receptors, and several orphan receptors [5]. RAR/RXR knockout mice develop different forms of heart defects dependent on the isoform knocked out, including heart malformations, defects in the conduction system, and heart failure [12]. Overexpression of RAR or RXR induces dilated cardiomyopathy, depresses cardiac function, and causes congestive heart failure [13].

We hypothesized that myocardial infarction evokes RA signaling and that RA regulated genes may have effects on postinfarction remodelling of the heart. A mouse model of in vivo infarction, RARE-luciferase reporter mice, as well as analysis of cardiac cells and tissue were used to test the hypothesis.

## Materials and Methods

### Animals

All experiments were performed according to the Declaration of Helsinki, and the experiments were approved by the Norwegian Animal Research Authority. Male C57BL/6 mice (25–30 g) or male retinoic acid response element luciferase reporter mice (RARE-Luc) (www.cgene.no) of the same weight were used for experiments as detailed below. Briefly, fertilized zygotes were obtained from superovulated C57BL/6 females mated to C57BL/6 males. Linearized DNA, containing three copies of RARE derived from the RAR-β2 attached to the luciferase firefly gene was injected into pronuclei of the zygotes. Zygotes were then transferred into the oviducts of pseudopregnant mice. All the mice used in these experiments were heterozygous for the transgene and back–crossed for more than six generations from founder mice to C57BL/6 [14].

### Induction of *in vivo* Myocardial Infarction

Mice were anesthetised with isoflurane and intubated, and surgery was performed as previously described [15]. Mechanical ventilation was used to maintain a respiratory rate of 135/min with pure oxygen mixed with 1.5–2.0% isoflurane, and a left-sided thoracotomy performed. The pericardium was cut open, and permanent ligation of the descending branch of the left coronary artery was made 1.5 mm under the tip of the left auricle using 8–0 silk suture. Sham operated mice underwent exactly the same procedures, except that the silk suture around the coronary artery was not tightened but rapidly withdrawn. Subsequently the intercostal space, muscles of the external thoracic wall and skin were sutured with 6/0 polyester. Extubation was performed upon spontaneous breathing. Animals were placed in a “mini intensive care unit” postoperatively, maintaining an environment of 30°C over night. All animals received 0.5 ml saline intraperitoneally prior to surgery to compensate for fluid loss and 0.1 mg/kg of buprenorphine hydrochloride (Temgesic, Schering-Plough AS) subcutaneously for analgesia. Postoperatively animals were inspected daily and Temgesic was administrated on the first postoperative day and later when animal behaviour suggested pain.

### *In vivo* Imaging of Cardiac Retinoic Acid Signalling

To evaluate in vivo cardiac activation of the retinoic acid receptors (RAR), luciferase reporter mice were subjected to myocardial infarction as described above or sham operated (n = 7–10 in each group). After shaving the ventral thoracoabdominal wall, mice were anesthetized with isoflurane (1.5–2.0%) and luciferase activity was measured in vivo serially after infarct induction using luciferin (Biosynth, Basel, Switzerland) and an IVIS 100 CCD camera (Xenogen, CA, USA). Luciferin (200 µl of 20 mg/mL; Biosynth, Basel, Switzerland) was injected intraperitoneally, and the thoracic region was imaged 12 minutes later (time span decided after pilot studies). Mice were imaged prior to infarct induction or sham operation, and followed up to six weeks after infarction. Data acquisition and quantification were done with the software Living Image (Xenogen). Light emission in the thoracic region was quantified as photons/s/cm^2^/sr. After the last *in vivo* imaging six weeks postinfarction, organs were harvested and imaged *ex vivo* to confirm that the signal was from the heart.

### Effect of Hypoxia Factor 1α (HIF-1α) Inhibition on Retinoic Acid Signalling *in vivo*

In order to investigate if retinoic acid signalling during ischemia was dependent on HIF-1α, RARE-luc reporter mice (n = 4 in each group) were injected i.p.with 25 mg/kg of the HIF-1α inhibitor PX-478 (conc. 250 ug/mL in 0.9% NaCl) or vehicle alone 2 hours prior to ligation of the coronary artery. The concentration of PX-478 was chosen based on experiments performed in similar in vivo models [16], [17]. PX-478 was injected daily up to 7 days postinfarction, and the RARE-luc activity was imaged daily 2 hours after administration of HIF-1α-inhibitor. Imaging of thoracic RARE luc was performed as described previously, and 7 days postinfarct hearts were extracted for ex vivo imaging. Infarct size was determined by TTC staining, and the bioluminescent signal was related to infarct size estimated in Photoshop.

### *Ex vivo* Imaging of Cardiac Retinoic Acid Signalling

Additional RARE-luc mice were subjected to myocardial infarction or sham operation (n = 7–8/group) for organ imaging one week postinfarction, when the *in vivo* signal was at its strongest. Mice were anesthetized with isoflurane, injected with luciferin, and hearts, lungs, thymus, liver, spleen, pancreas and epidydimal white adipose tissue were surgically removed. Organs were placed in a petridish and the luciferase signal measured and quantified in the CCD camera.

### Heart Sampling for Gene Expression

In another series of experiments, C57BL/6 wild type mice were subjected to *in vivo* induced infarction or sham operation as described above, and hearts were sampled serially postinfarction (after 24 hours, 1 week, 4 weeks, and 6 weeks) for RNA extraction and amplification of RA-regulated target genes. A control group without infarction was added (n = 6 to 8 in each group at each time point).

### Western Blot

Protein expression was investigated using western blot technic, as described in more detail in online supplement (Methods S3). Tissue samples from infarcted and sham operated hearts one week after induction of infarction (n = 7 of each) were homogenized in RIPA lysis buffer (Millipore, Temecula, CA, USA, supplemented with Halt™ Protease & Phosphatase Inhibitor Cocktail (Thermo Scientific), and 40 µg protein of each was separated on SDS-polyacrylamide gels (Criteron, BIoRad) and transferred onto nitrocellulose membranes (Amersham Biosciences Europe, Germany). Membranes were incubated in TBS +0.1% Tween-20, first with 5% skimmed milk powder, then over-night at 4°C with goat polyclonal anti-RBP-1 (1∶2500; Cat # PAB6754, Abnova) or rabbit polyclonal anti-ALDH1A2 (1∶1000; Cat # 13951-1-AP, Proteintech, Manchester, UK). HRP-conjugated secondary antibodies were used to vizualize protein bands on photographic film by chemiluminescence (ECLplus; Pierce, Rockford, IL, USA). Scanned images of exposed films and membranes stained with Ponceau solution for protein loading evaluation, were analyzed using the Image Quant software, and signal intensity of target protein bands were related to the Ponceau staining to account for protein loading and blotting efficiency.

### Measurement of Retinoic Acid Metabolites

Infarcted or sham operated RARE luciferase reporter hearts used for *ex vivo* luciferase activity were also used for measuring RA metabolites 1 week postinfarction. The infarcted zone of the left ventricle was dissected from periinfarcted tissue and samples were separately snap frozen in liquid nitrogen. The samples from sham operated hearts were uses for comparison. Heart samples were homogenized in ice-cold phosphate-buffered saline with a motorized homogenizer (Pro Scientific, Inc., Oxford, CT), and retinoids were extracted with ice-cold acetonitrile containing ^13^C-labeled atRA as an internal standard (IS). The concentration of the IS was 10 ng/mL. An aliquot was injected into a 4000 Q TRAP LC-MS/MS instrument with APCI ionization (Applied Biosystems, California). The liquid chromatography-mass spectrometry conditions were as described previously [18], except that the separating column was an ABZ Plus (75 by 3 mm [inner diameter], 3-µm particles; Supelco, Pennsylvania). The entire procedure was performed under red light. Calibration graphs were constructed by linear least-squares regression analysis, plotting peak area ratios of the analyte concentration and the IS against the corresponding concentrations. Quantification was carried out by interpolation and linear least-squares regression [18].

### Isolation and Culture of Adult Mouse Cardiomyocytes and Cardiofibroblasts

Adult mouse cardiomyocytes and cardiofibroblasts were isolated from hearts from 5 infarcted and 5 sham operated RARE-luc reporter mice using the method described by [19]. Cells were isolated for evaluation of RARE reporter gene activity on the 7^th^ postoperative day as described in more detail in online supplement (Methods S1). The infarcted and periinfarcted zone of the left ventricle were dissected and used for cell isolation in parallel with extracting cells from sham operated hearts. Hearts were perfused with digestion buffer containing Collagenase II (Worthington Biochemical, Lakewood, NJ), and mechanically disrupted. Cardiomyocytes were separated from cardiofibroblasts by serial centrifugations [19]. Cardiofibroblasts were collected from the first centrifugation, transferred to a separate tube, resuspended, and plated [20]. Cardiomyocytes were resuspended laminin (BD- biosciences) coated six-well plates (see online supplement). The cells were incubated for 3 hours at 37°C in an atmosphere supplied with 2 or 5% CO_2,_ before RARE reporter gene activity was measured using the same CCD camera as for *in vivo/ex vivo* imaging after adding luciferin (100 µL of 20 mg/mL). Finally, cells were harvested for mRNA isolation and stored at –80°C.

### RNA Extraction and Real-time q-PCR

RNA was extracted using RNeasy Mini Kit (QIAGEN inc.) with an additional phenol-chloroform extraction step and in-column DNase treatment (QIAGEN). Random hexamers for priming (3 min at 70°C) were used for reverse transcription of 1 µg of RNA from whole heart extracts and 200 ng of RNA from isolated cardiomyocytes and cardiac fibroblasts. Reverse transcription was followed by a modified First Strand cDNA Synthesis Protocol with Superscript III (Invitrogen) and RNasin (Promega) enzymes and cDNAs were amplified using real-time PCR. Primers for RAR target genes and endogenous control rpl32 were designed with Primer Express 3.0 software and are shown in online supplement (Table S1). SYBR green (Applied Biosystems, Foster City, CA, USA) was used for detection. A predesigned Real-Time PCR primer pair and probe (TaqMan Gene Expression Assays, Applied Biosystems) for detection of 18S rRNA was used as endogenous control for whole heart extracts, whereas rpl32 was used as endogenous control for cardiomyocytes and cardiofibroblasts after performing pilot studies on endogenous control suitability. For details of PCR reaction, please see supplementary material online (Methods S2).

### *In vitro* Evaluation of atRA Effects on Cardiofibroblast Proliferation

Cardiofibroblasts were isolated from 4 healthy C57BL/6 mouse hearts. Cells were incubated up to 96 h in medium supplemented with 1 µM atRA dissolved in ethanol (Sigma Aldrich; St. Louis, USA) and 10 µM EdU (5-ethynyl-2′-deoxyuridine; nucleoside analogue to thymidine which is incorporated into DNA during active DNA synthesis). atRA and EdU supplemented medium was changed every 24 h. Control experiments were performed using ethanol only. Cells were harvested for proliferation evaluation by EdU incorporation by flow cytometry as described in more detail in the online supplement (Methods S4).

### Statistics

Repeated measurements of luminescence activity postoperatively in the thoracic region were evaluated using repeated measures ANOVA. The non-parametric Mann–Whitney *U* test was used to compare gene expression data, luminescence between explanted organs and isolated cells, where a non-Gaussian distribution was assumed. Data are presented as mean ± SD. Differences were considered significant when P<0.05 and a tendency was regarded when P was 0.05–0.08.

## Results

### Thoracic Retinoic Acid Signalling is Activated Early after Myocardial Infarction

**Figure 1:**
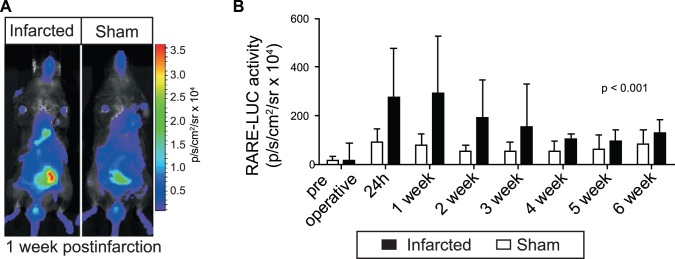
*In vivo* imaging of cardiac retinoic acid signalling after myocardial infarction. To evaluate *in vivo* cardiac activation of the retinoic acid receptor RAR, RARE-luciferase reporter mice were subjected to myocardial infarction through permanent coronary artery ligation or sham operation (n = 7–10 of each). *In vivo* luciferase activity was measured in a CCD camera after injection of luciferin preoperatively and serially after infarction. A representative image of an infarcted and a sham-operated mouse is shown on the left (**A**). On the right, the digitized luciferase signal is quantified and shown as bar graphs (**B**; mean±SD).

RAR luciferase reporter mice were used to determine if RA signalling measured as luminescence was activated in the heart after induced infarction through *in vivo* imaging. The RAR reporter activity was increased in the thoracic region of infarcted animals, indicating activation in the heart. Infarction-induced thoracic RAR activity reached the highest level at the end of the first postoperative week and gradually declined, but remained higher than sham throughout the six weeks observation period (Fig. 1).

### Retinoic Acid Signalling is Independent of Hypoxia-inducible Factor 1α

RARE-luc activity was measured and quantified in RARE luc reporter mice treated daily with the HIF-1α inhibitor PX-478 or saline in conjunction with infarct induction. No differences were found between groups during 1 week observation (Figure S1).

### Retinoic Acid Signalling was Increased Specifically in the Infarcted Heart

**Figure 2:**
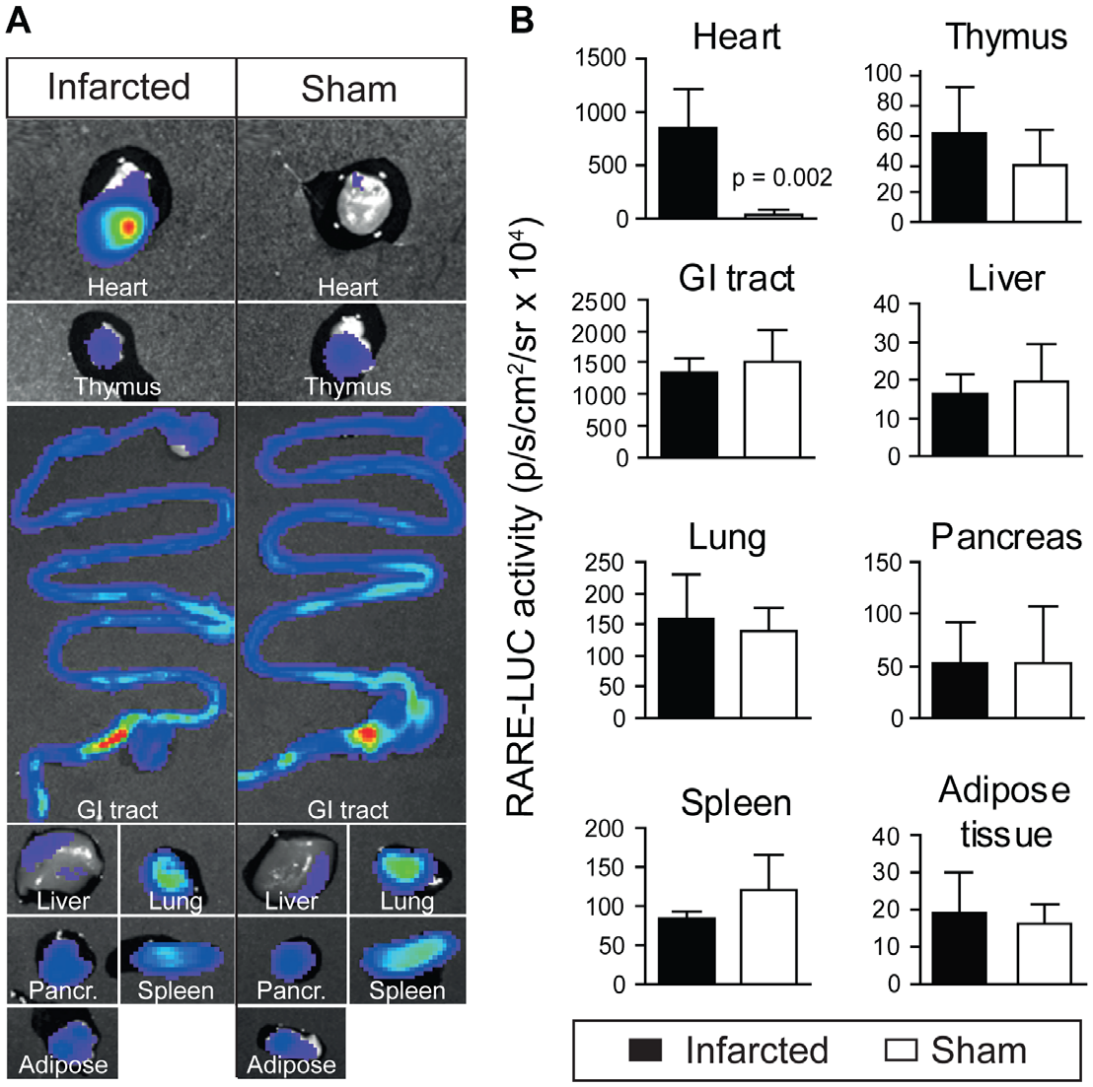
*Ex vivo* imaging of cardiac retinoic acid signalling after myocardial infarction. To verify that the signal in the thoracic region detected *in vivo* originated from hearts, organs were harvested one week after infarction or sham operation following in vivo luciferin injection in RARE-luciferase reporter mice. Organs were placed in a petridish and the luminescence signal quantified. A representative image of one single experiment is shown on the left (**A**), where hearts, thymus, gastrointestinal tract (GI-tract), liver, lungs, pancreas, spleen and epididymal white adipose tissue from one infarcted and one sham operated animal are shown. When the signal was quantified from 6 independent experiments, the luminescence was exclusively increased in infarcted hearts, and not in sham operated hearts or any other organs (**B**). Data are shown as mean±SD.

Since *in vivo* signalling in the thoracic region could be of non-cardiac origin, selected organs were harvested and imaged in the CCD camera to verify the source of signal. One week postinfarction, when the *in vivo* signalling peaked, the RAR reporter activity was higher in infarcted hearts than in sham operated hearts (Fig. 2). There were no differences in luminescence between groups in any of the other imaged organs (Fig. 2). RAR reporter activity remained higher in infarcted hearts than in sham operated hearts six weeks postinfarct (results not shown).

### Increased *in vivo* Expression of RA Target Genes

**Figure 3:**
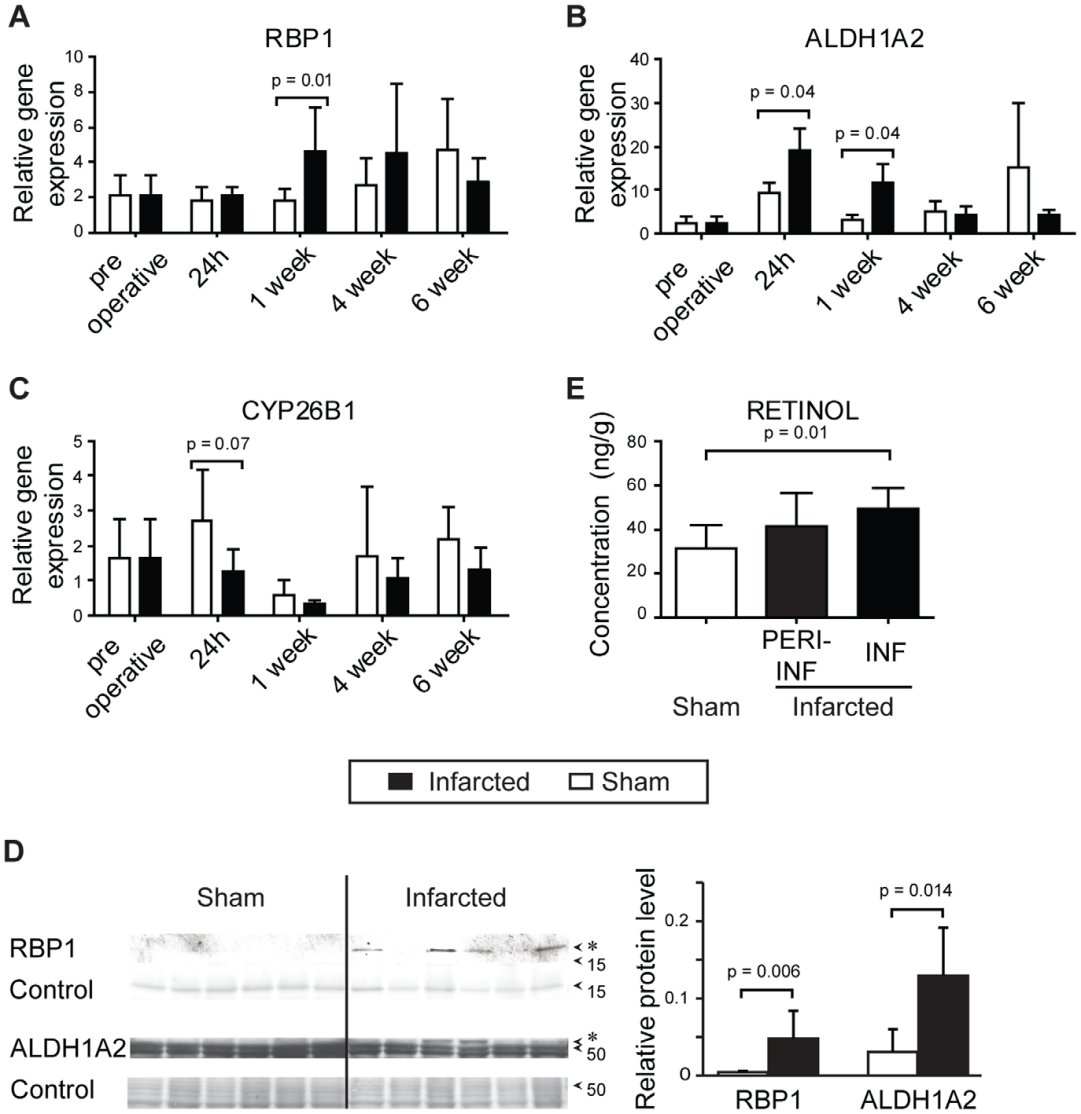
Expression of retinoic acid target genes and endogenous metabolites after infarction. Wild type C57Bl6 mice were subjected to *in vivo* ligation of the left coronary artery or sham surgery, and hearts were sampled serially for RNA extraction and amplification of retinoic acid target genes with real time PCR, or proteins were extracted for western blotting. Hearts were also sampled from mice without any surgery (preoperative). Gene expression of retinol binding protein 1 (RBP1) (**A**), aldehyde dehydrogenase 1A2 (ALDH1A2) (**B**), and cytochrome p45026B1 (CYP26B1) are shown (**C**). (n = 6–8 in each group at each time point). **D)** Representative western blot analysis of retinoic acid transporting and metabolizing proteins one week after induction of infarction or sham operation. Ponceau solution was used as protein loading (control). Histograms show the relative density of RBP1 at 16 kDa and ALDH1A2 at 53–57 kDa in infarcted and sham operated hearts. **E)** Endogenous retinoic acid metabolite concentrations were evaluated by triple-stage liquid chromatography/tandem mass spectrometry one week after induction of myocardial infarction. The infarcted zone of the left ventricle (INF), the periinfarcted zone (PERIINF) and left ventricles from sham operated hearts (SHAM) were investigated (n = 6). Data are shown as mean±SD.

Other mice were subjected to in vivo infarction or sham operation for sampling of hearts for RNA extraction and amplification with real time PCR 24 hours, 1, 4 and 6 weeks postinfarct. Expression of RBP1 mRNA was increased one week postinfarction (Fig. 3a). ALDH1A2 was increased compared with sham 24 hours and 1 week postinfarction (Fig. 3b). CYP26B1 tended to be downregulated early post infarction and 4 weeks later (Fig. 3c). STRA6, CRABP1, CRABP2 and RAR α,β,γ, were unchanged (data not shown).

### Increased Expression of RA Transporting and Metabolising Proteins

To investigate whether retinoic acid transporting and metabolizing proteins are induced after myocardial infarction, we extracted proteins from infarcted and sham operated hearts one week after coronary artery ligation for western blotting with antibodies against RBP1 and ALDH1A2. Protein expression of RBP1 and ALDH1A2 were significantly upregulated in infarcted hearts one week after infarct, but not in sham operated hearts (Fig. 3d).

### The Infarcted Myocardium Accumulates Retinol

Investigation of endogenous RA metabolite concentrations in infarcted and sham operated RARE-luc hearts one week later by triple-stage liquid chromatography/tandem mass spectrometry showed accumulation of retinol in infarcted hearts. Increased levels of retinol were found in the infarcted tissue itself and not in the periinfarct zone or in sham operated hearts (Fig. 3e). Retinal, 13-cis RA or atRA were unchanged (data not shown).

### Cardiofibroblasts are the Major Source of Luminescence in the Infarcted Heart

**Figure 4:**
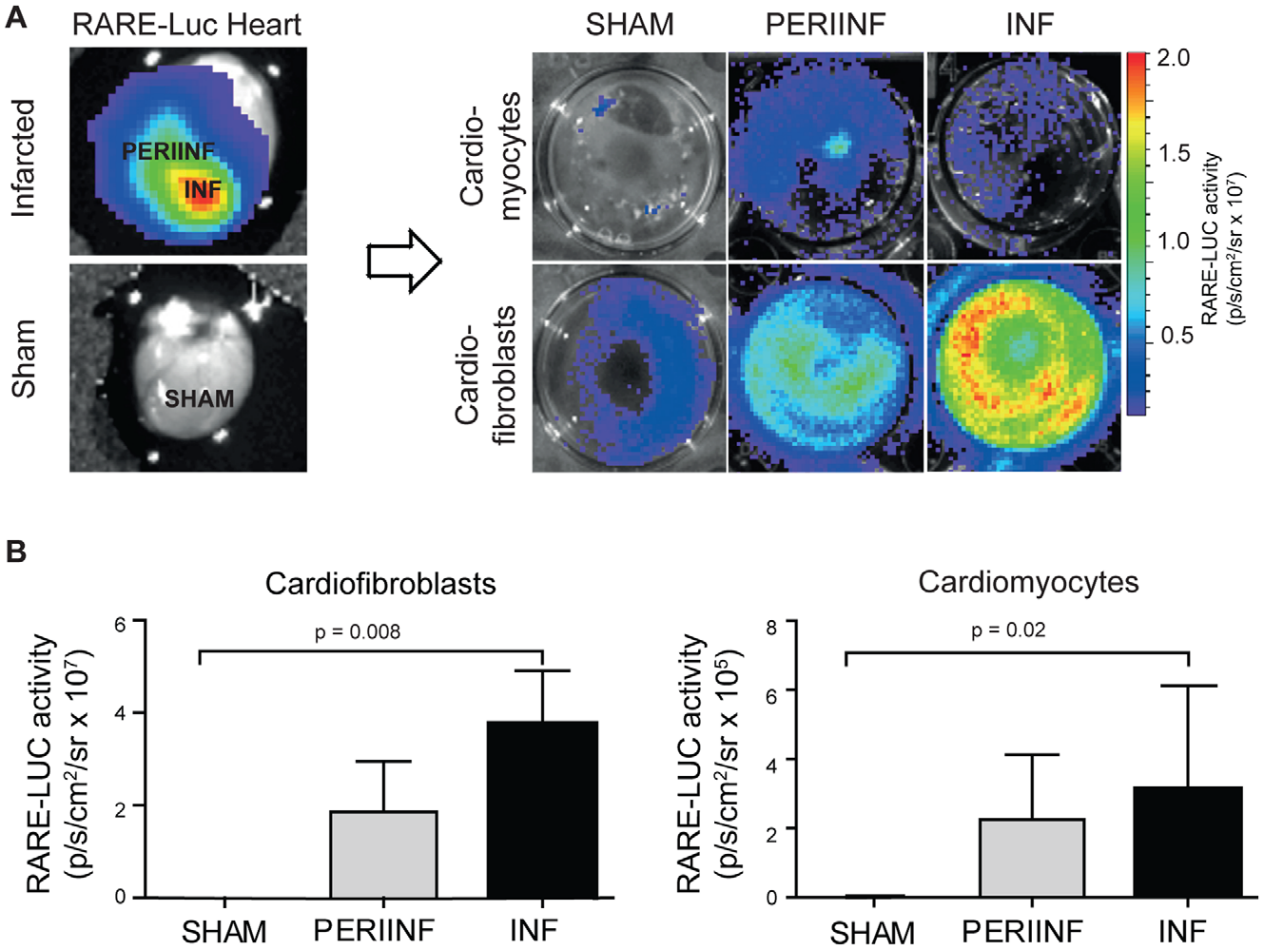
*In vitro* imaging of retinoic acid signaling after myocardial infarction. Cardiomyocytes and cardiofibroblasts were isolated from left ventricular tissue from RARE-luciferase reporter hearts one week after myocardial infarction or sham operation (SHAM). Cells were isolated from the infarct (INF) and periinfarct zone (PERIINF). After plating for three hours, non-viable cells were removed and luciferin was added for imaging. The upper panel shows a representative image of one experiment (**A**). The lower panel shows mean±SD of n = 5 experiments in each group (**B**). Note that the Y-axis labelling is different.

Cardiomyocytes and cardiofibroblasts were isolated from the infarcted area and the periinfarct zone of infarcted hearts, or from sham operated RARE-luc reporter hearts. Luciferase activity was measured after plating of cells. Fibroblasts isolated from the infarcted zone of infarcted hearts had increased luminescence compared with fibroblasts from sham operated hearts (Fig. 4). Luminescence was similar in cells harvested from the periinfarcted zone and the infarct zone. Hardly any luciferase activity was detected in cardiomyocytes of sham operated mice, while the signal intensity increased after infarction with no differences between infarcted and periinfarcted cells (Fig. 4).

### Gene Expression of RA Target Genes in Infarct Zone is Higher in Cardiofibroblasts than in Cardiomyocytes

**Figure 5:**
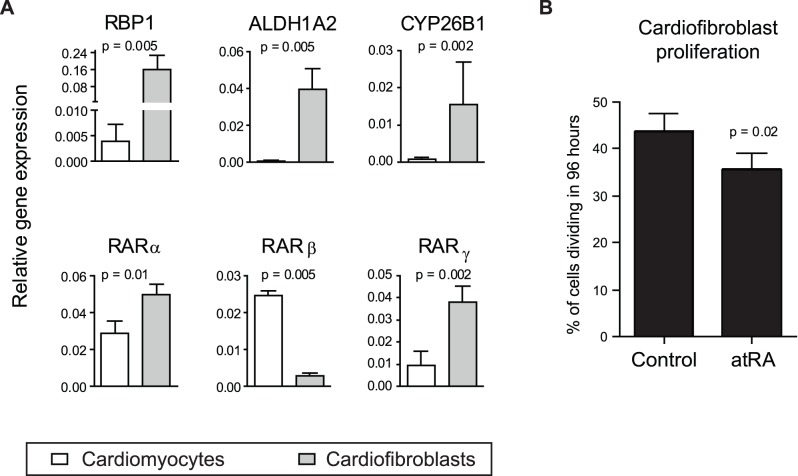
Expression of retinoic acid target genes in the infarct zone and effects on cardiofibroblast proliferation. **A)** Gene expression of retinoic acid target genes in cardiofibroblast (CF) and cardiomyocytes (CM) isolated from left ventricles of infarcted RAR-luciferase reporter hearts 1 week after infarction. RNA was extracted from cells from the infarcted zone and amplified with real time PCR using primers specific for retinol binding protein 1 (RBP1), cytochrome P450 26B1 (CYP26B1), aldehyde dehydrogenase 1A2 (ALDH1A2), and retinoic acid receptors alpha, beta and gamma (RAR α,β,γ). The figure shows test gene expression relative to expression of rpl32, which was similar in both cell types. Data are mean±SD of n = 5 experiments in each group. **B)** Cardiofibroblasts were isolated from C57BL6 hearts and cultured in medium supplemented with 1 µM all-trans retinoic acid (atRA) and 10 µM EdU, as indicator of cell division. After 96 hours, EdU incorporation was evaluated by flow cytometry. Data are presented as mean±SD of n = 4 in each group.

The cells isolated from the infarct zone of hearts used for RARE-luc activation were used for RNA extraction and amplification of RA target genes through real time PCR. The CT value of the endogenous control rpl32 was similar in both cell types. In accordance with increased luciferase activity in cardiofibroblasts, gene expression of RBP1, CYP26B1, ALDH1A2, RARα and RARγ was higher in cardiofibroblasts than in cardiomyocytes. RARβ was the only examined gene which was more highly expressed in cardiomyocytes (Fig. 5a).

### *In vitro* Evaluation of atRA Effects on Cardiofibroblast Proliferation

Incubation of cardiofibroblasts with 1 µM atRA inhibited cell proliferation in vitro when EdU incorporation was evaluated 96 hours later by flow cytometry (Fig. 5b).

## Discussion

The main finding of the present study was that the RA signalling pathway was activated in the mouse heart with permanent coronary artery ligation. Using a reporter mouse with firefly luciferase coupled to RA response element, a dramatic increase of luciferase signal was found *in vivo* in the thoracic region of mice with induced infarction, peaking the first postoperative week. The signal was verified to originate exclusively from the heart by *ex vivo* organ imaging, with maximal signal emission in the infarcted zone. This was accompanied by increased cardiac expression of genes and proteins involved in regulation of RA metabolism RBP1 and ALDH1A2, while CYP26B1 mRNA, an endogenous enzyme responsible for atRA degradation, was downregulated. Increased gene and protein expression of retinol transporting proteins coincided with accumulation of retinol in infarcted hearts. Cardiofibroblasts and cardiomyocytes had RARE-Luc activation postinfarct, and increased fibroblast expression of the RA target genes RBP1, ALDH1A2, CYP26B1, RARα and RARγ, while RARβ was more highly expressed in cardiomyocytes. AtRA inhibited proliferation of cardiofibroblasts *in vitro*. Anti-proliferative effects of atRA in cardiac fibroblasts is clinically appealing, as dietary RA supplementation is easy to perform in humans with myocardial infarction. The antiproliferative properties of atRA are already used in the clinics as treatment of different leukemias.

To our knowledge, no previous studies have reported activation of retinoic acid signalling in the heart in conjunction with myocardial ischemia and remodelling. It seems to be independent of hypoxia*-*inducible factor 1 alpha (Fig. S1). The increased RARE-luc activity as well as increased expression of retinol transporting genes and proteins could lead to cardiac accumulation of retinoic acid postinfarction. This hypothesis was supported by the finding of altered content of retinol in postinfarcted hearts in the current study. Our findings concur with one previous study investigating RA content in postischemic hearts. Palace and coworkers injected postinfarcted rats with radiolabelled vitamin A. Radiolabelled vitamin A was increased in hearts and plasma of rats with myocardial infarction, while labelled kidney and liver retinol was lower in those animals. The authors speculated that it would be beneficial for an ischemic heart to increase antioxidant content. However, accumulation of exogenously administrated atRA does not necessarily reflect the role of endogenous atRA postinfarction.

Activation of RARs in the post ischemic heart may play a role in regulation of damage and repair. We did not address the role of retinoic acid *in vivo* in the present study. This has previously been done by others: Rats with tissue insufficiency of vitamin A had spontaneous cardiac remodelling and ventricular dysfunction [7]. When myocardial infarction was induced in rats with tissue vitamin A deficiency, adverse left ventricular remodelling was intensified [21]. Supplementing rats with retinoic acid in a model of tobacco smoke-induced left ventricular remodelling could prevent remodelling [22]. Retinoic acid supplementation could also prevent remodelling induced by left coronary artery ligation [8].

The heart consists of several cell types, where cardiofibroblasts and cardiomyocytes are the most abundant. To explore the relation between cell types and RAR response, cardiomyocytes and cardiofibroblasts were isolated from infarcted hearts. Interestingly, cardiofibroblasts isolated from the infarcted or periinfarcted zones had increased luminescence compared to fibroblasts from sham operated hearts. RAR luciferase activity increases also in cardiomyocytes from infarcted and periinfarcted tissue. Fibroblasts had a higher increase of RA target genes than myocytes. Unlike the cardiomyocytes, which have low stem cell potential, cardiofibroblasts are actively dividing and differentiating.

Cardiofibroblasts stimulated with atRA had reduced cell proliferation, supporting a potential beneficial role of atRA during remodelling. Others have studied the effect of atRA on proliferation of neonatal cardiac cells: The hypertrophic response evoked by cyclic stretching of cardiomyocytes was inhibited by atRA [23]. Wu and collaborators used atRA to counteract hypertrophic responses to endothelin in neonatal cardiomyocytes [24]. In neonatal cardiofibroblasts, Wang and co-workers found that atRA dose-dependently reduced angiotensin-induced hyperplasia, and reduced the total cell protein content [9]. We confirm that anti-proliferative effects of atRA applies to adult cardiofibroblasts *in vitro.*

### Conclusions

All-*trans* retinoic acid (atRA) is used in therapy and prevention of many proliferative diseases such as prostate cancer or acute promyelocytic leukemia [25]. Because of their important role as regulators of cellular growth, differentiation, morphogenesis and metabolism, retinoic acid metabolites may also be of use to modify the proliferative response of cardiac cells in the process of remodelling. However, their mechanism of action and potential use as an anti-remodelling intervention in humans remain to be investigated.

## Supporting Information

Figure S1 Retinoic acid signalling is independent of hypoxia-inducible factor 1α (HIF-1α). RARE-luc activity in thorax was measured and quantified in mice treated daily with the HIF-1α inhibitor PX-478 or saline in conjunction with infarct induction. No differences were found between groups. Data are mean values of n = 4 in each group.
(EPS)

Table S1 Primer sequences of the investigated genes.
(XLS)

Methods S1 Isolation and culture of adult mouse cardiomyocytes and cardiofibroblasts.
(DOCX)

Methods S2 Evaluation of RA target genes expression using Real-time polymerase chain reaction.
(DOCX)

Methods S3 Evaluation of RA transporting and metabolizing proteins expression using western blot.
(DOCX)

Methods S4 *In vitro* evaluation of atRA effects on cardiofibroblast proliferation.
(DOCX)
