# Supplementary material for: Retinoic Acid Signalling Is Activated in the Postischemic Heart and May Influence Remodelling
Source: PLoS One. 2012 Sep 28;7(9):e44740. doi: 10.1371/journal.pone.0044740 (PMC3460971; doi:10.1371/journal.pone.0044740)
Supplement: Methods S1 — Isolation and culture of adult mouse cardiomyocytes and cardiofibroblasts. (DOCX) [file pone.0044740.s003.docx]

Adult mouse cardiomyocytes and cardiofibroblasts were isolated from hearts from 5 infarcted and 5 sham operated RARE-luc reporter mice using the method described by O'Connell [[23](#_ENREF_23)]. Cells were isolated for evaluation of RARE reporter gene activity on the 7^th^ postoperative day. Hearts were explanted, cannulated through the aorta and purged of blood with a perfusion buffer containing: NaCl (120 mM); KCl (15 mM); KH_2_PO_4_ (0.6 mM); Na_2_HPO_4_ (0.6 mM); MgSO_4_ (1.2 mM); Na-HEPES (10 mM); NaHCO_3_ (4.6 mM); Taurine (30 mM); Butanedione Monoxime (10 mM); Glucose (5.5 mM). The infarcted and periinfarcted zone of the left ventricle were dissected and used for cell isolation in parallel with extracting cells from sham operated hearts. Hearts were perfused with digestion buffer containing 2.4 mg/ml Collagenase II (Worthington Biochemical, Lakewood, NJ). Digested hearts were mechanically disrupted and suspended in perfusion buffer supplemented with 12.5 µM CaCl_2_ and 5% fetal calf serum (FCS). Cardiomyocytes were separated from cardiofibroblasts by serial centrifugations as described elsewhere [[23](#_ENREF_23)]. Cardiofibroblasts were preserved in the supernatant from the first centrifugation and transferred to a separate tube for resuspension in Minimum Essential Medium with Hank’s balanced salt solution (Gibco–BRL) and additives as described earlier [[24](#_ENREF_24)]. The suspension was plated on non-coated six-well plates. Cardiomyocytes were resuspended in Minimum Essential Medium with Hank’s balanced salt solution with additives and plated on laminin (BD- biosciences) coated six-well plates. The cells were incubated for 3 hours at 37 °C in an atmosphere supplied with 2 or 5 % CO_2,_ before RARE reporter gene activity was measured using the same CCD camera as for *in vivo/ex vivo* imaging after adding luciferin (100 μL of 20 mg/ mL) to each of the wells. Finally, cells isolated from infarct zone, periinfarct zone and sham operated hearts were harvested for mRNA isolation in 600 µL RLT buffer (Qiagen), using a cell scraper, snap-frozen in liquid N_2_, and stored at – 80 °C.
