# Supplementary material for: Retinoic Acid Signalling Is Activated in the Postischemic Heart and May Influence Remodelling
Source: PLoS One. 2012 Sep 28;7(9):e44740. doi: 10.1371/journal.pone.0044740 (PMC3460971; doi:10.1371/journal.pone.0044740)
Supplement: Methods S2 — Evaluation of RA target genes expression using Real-time polymerase chain reaction. (DOCX) [file pone.0044740.s004.docx]

PCR-reactions took place in 96-well plates using 1 μL of cDNA, 10 μL of Power SYBR Green PCR Master Mix (Applied Biosystems), and primers at a final concentration of 900 nM in a total volume of 20 μL. The PCR reaction had the standard amplification scheme: one cycle of 2 minutes at 50°C (AmpErase UNG activation), one cycle of 10 minutes at 95°C (Gold AmpliTaq activation, AmpErase UNG inactivation), followed by 40 cycles of denaturation for 15 seconds at 95°C and annealing/extension for 1 minute at 60°C in a ABI 7900 HT Sequence Detection System (Applied Biosystems). CT cycle values were correlated to a standard curve. The resulting mRNA levels relative to the 18S rRNA or rpl32 were calculated according to the standard formula 2-ΔΔCT, where ΔΔCT= (CTTarget_sample - CTendogenous control _sample)-(CTTarget_calibrator –CTendogenous control_calibrator) as described by user bulletin #2; Applied Biosystems.
