# Supplementary material for: Retinoic Acid Signalling Is Activated in the Postischemic Heart and May Influence Remodelling
Source: PLoS One. 2012 Sep 28;7(9):e44740. doi: 10.1371/journal.pone.0044740 (PMC3460971; doi:10.1371/journal.pone.0044740)
Supplement: Methods S3 — Evaluation of RA transporting and metabolizing proteins expression using western blot. (DOCX) [file pone.0044740.s005.docx]

Proteins were extracted from infarcted and sham operated hearts one week after induction of infarction. Heart samples were separately homogenized using rotor-stator homogenizer (IKA works, Germany) in RIPA lysis buffer (Millipore, Temecula, CA, USA, supplemented with Halt™ Protease & Phosphatase Inhibitor Cocktail (Thermo Scientific). Samples were centrifuged at 4 °C for 5 min (18000 g), and the supernatant was collected. Protein concentration of the samples were determined by the BCA assay (Pierce, USA), and 40 µg protein was loaded per lane on 15 % and 4-20 % gradient SDS-polyacrylamide gels (Criteron, BIoRad) for RBP-1 (16 kDa) and ALDH1A2 (53-57 kDa) detection, respectively. After electrophoresis proteins were transferred onto nitrocellulose membranes (Amersham Biosciences Europe, Germany), which were blocked 90 minutes in TBSS washing buffer (TBS + 0.1 % Tween-20) supplemented with 5 % skimmed milk powder, prior to incubation over-night at 4 °C with goat polyclonal anti-RBP-1 (1:2500; Cat # PAB6754, Abnova) or rabbit polyclonal anti-ALDH1A2 (1:1000; Cat # 13951-1-AP, Proteintech, Manchester, UK) antibodies and washed Thereafter, the blots were incubated with HRP-conjugated rabbit anti-goat or goat anti-rabbit secondary antibodies (1:10000 or 1:5000, respectively; Southern Biotech, England), washed, and protein bands vizualized on photographic film (Amersham Hyperfilm ECL, GE healthcare) by chemiluminescence (ECLplus; Pierce, Rockford, IL, USA). The exposed films (60 minutes) and membranes stained in Ponceau protein dye solution were scanned with CanonScan Lide 35 scanner (Epson Perfection V700). Images were analyzed using the Image Quant software, and signal intensity of target protein bands were related to the Ponceau staining to account for protein loading and blotting efficiency.
