# Supplementary material for: Retinoic Acid Signalling Is Activated in the Postischemic Heart and May Influence Remodelling
Source: PLoS One. 2012 Sep 28;7(9):e44740. doi: 10.1371/journal.pone.0044740 (PMC3460971; doi:10.1371/journal.pone.0044740)
Supplement: Methods S4 — In vitro evaluation of atRA effects on cardiofibroblast proliferation. (DOCX) [file pone.0044740.s006.docx]

Cardiofibroblasts were isolated from 4 healthy C57BL/6 mouse hearts. Cells were incubated up to 96 h in medium supplemented with 1 µM atRA dissolved in ethanol (Sigma Aldrich; St. Louis, USA) and 10 μM EdU (5-ethynyl-2´-deoxyuridine; nucleoside analogue to thymidine which is incorporated into DNA during active DNA synthesis). atRA and EdU supplemented medium was changed every 24 h. Control experiments were performed using ethanol only. Cells were harvested for proliferation evaluation by EdU incorporation. Cardiofibroblast proliferation was determined using the Click-iT™ cell proliferation kit for flow cytometry (Invitrogen, C-10418). In brief, cells were incubated in 1 mL accutase allowing cells to detach for 5-10 minutes in the 37 °C incubator; fixed with 100 μL 4% paraformaldehyde for 15 minutes at room temperature; permeabilized with 100 μL of Click-iT™ saponin-based permeabilization buffer; resuspended in 0.5 mL Click-iT™ reaction cocktail; resuspended in PBS; and 1000 events were analyzed using a LSR II flow cytometer (BD-biosciences). Pacific Blue™ was detected with logarithmic amplification using 406 nm excitation with violet (450/50 nm) emission filter. Side- and forward-scatter was monitored and used to exclude debris and doublets.
